# Performance Assessment of Pneumatic-Driven Automatic Valves to Improve Pipeline Fault Detection Procedure by Fast Transient Tests

**DOI:** 10.3390/s24061825

**Published:** 2024-03-12

**Authors:** Francesco Castellani, Caterina Capponi, Bruno Brunone, Matteo Vedovelli, Silvia Meniconi

**Affiliations:** 1 Department of Engineering, The University of Perugia, 06125 Perugia, Italy; matteo.vedovelli@unipg.it; 2 Department of Civil and Environmental Engineering, The University of Perugia, 06125 Perugia, Italy; caterina.capponi@unipg.it (C.C.); bruno.brunone@unipg.it (B.B.); silvia.meniconi@unipg.it (S.M.)

**Keywords:** valve hydraulic behaviour, fault detection, pipelines, transients, water hammer

## Abstract

The use of fast transients for fault detection in long transmission networks makes the generation of controlled transients crucial. In order to maximise the information that can be extracted from the measured pressure time history (pressure signal), the transients must meet certain requirements. In particular, the manoeuvre that generates the transient must be fast and repeatable, and must produce a pressure wave that is as sharp as possible, without spurious pressure oscillations. This implies the use of small-diameter valves and often pneumatically operated automatic valves. In the present work, experimental transient tests are carried out at the Water Engineering Laboratory (WEL) of the University of Perugia using a butterfly valve and a ball pneumatic-driven valve to generate pressure waves in a pressurised copper pipe. A camera is used to monitor the valve displacement, while the pressure is measured by a pressure transducer close to the downstream end of the pipe where the pneumatic valve is installed. The experimental data are analysed to characterise the valve performance and to compare the two geometries in terms of valve closing dynamics, the sharpness of the generated pressure wave and the stability of the pressure time history. The present work demonstrates how the proposed approach can be very effective in easily characterising the transient dynamics.

## 1. Introduction

Until a few years ago, systematic measures to reduce Non-Revenue Water (NRW) in pressurised pipe systems focused mainly on fault (leak) detection techniques in water distribution networks (WDNs) (e.g., [1,2,3]). However, transmission mains (TMs) were often negligently excluded from fault (leak) detection programmes [4]. It is only recently that water companies have become more aware of the risks posed by leaks in TMs. In fact, TMs carry a large part—in most cases, all—of the discharge to be supplied and so even a small percentage of losses means a large volume of wasted water. This has led to the need to carry out fault detection campaigns in TMs and to identify appropriate and reliable technologies. In fact, TMs have fewer appurtenances and are buried deeper and in less accessible locations than WDNs. Most inspection technologies for TMs are of an in-line type (e.g., [5], with sensors inserted into the pipeline [6,7,8,9]). Specifically, there are two main types of techniques: (i) acoustic sensors with a wireless communication network (e.g., echologics.com)); and (ii) free-swimming sensors (e.g., xylem.com). The first technique requires probes to be placed in dedicated manholes at a maximum distance of 1 km. This requires considerable adaptation to existing pipelines and increases the construction costs of new ones. The second technique uses special probes (e.g., SmartBall and Sahara) to explore the pipeline [10,11]. To facilitate the insertion/retrieval of these probes, special devices have to be built, and the pressure has to be reduced. As an alternative to in-line techniques, Transient Test-Based Techniques (TTBTs) have attracted attention in recent decades due to their advantages, in particular: (i) the short duration of the tests, (ii) the need for pressure measurements only, (iii) the fact that they allow the detection of insidious defects that do not give any external sign (e.g., partial blockage [12]) and (iv) the relatively low cost (e.g., [13,14,15,16,17,18]). In addition, the diagnosis procedure can be carried out by the water company’s technicians with complete autonomy and at very low cost whenever they deem it necessary, after appropriate training on the job. After laboratory verification [19,20], TTBTs have been successfully used in real pipe systems characterised by large diameters and lengths (e.g., [21,22]). More specifically, TTBTs consist of the generation of a controlled (i.e., repeatable and safe) pressure wave that propagates into the pipe system being tested. As the pressure wave propagates, if it encounters a fault (e.g., leak, partial blockage, wall deterioration) or a singularity (e.g., series connection, partially closed in-line valve), it is partly reflected back and partly transmitted forward. The amplitude and sign of such waves depend on the specific fault/singularity. For example, when a pressure wave interacts with a leak, a negative reflected pressure wave travels back with an amplitude proportional to the size of the leak and inversely proportional to the pressure at the leak (e.g., [23,24,25,26,27,28]). The analysis of the pressure time history (pressure signal) measured during the transient test at a properly arranged measurement section provides useful information for pipe diagnosis. In fact, the aim of any fault detection procedure is to locate the anomaly and identify its type and severity. Within TTBTs, both short and long period pressure signal analysis can be used to provide useful diagnostic information [15]. In the former, attention is focused on the first phase of the transient event, and the pressure variations due to the initial interactions of the generated pressure wave with the fault/singularity are studied. In the latter, the overall damping of the pressure peaks is considered and evaluated in both time and frequency domains [29].

The analysis of the acquired pressure signals can be performed using Inverse Transient Analysis (ITA) (e.g., [30,31,32]). The ITA approach is based on the comparison of numerical and experimental pressure signals with the aim of determining one or more parameters (e.g., fault characteristics) that minimise the difference between them by using optimisation algorithms (e.g., [33]). Another option is the Direct Transient Analysis (DTA), which does not involve the use of numerical models. Rather, it is based on the study of the experimental pressure signal, in which pressure variations (i.e., the reflected and transmitted pressure waves) are identified and their consistency with information about the pipe system under investigation is assessed. In this way, any anomaly (due to a fault) with respect to the expected behaviour is detected and characterised (e.g., [34,35,36,37,38,39]). In this respect, any time domain analysis based only on the damping of pressure peaks may lead to ambiguity [40]. In fact, it has been shown by numerical and laboratory [41] experiments that two leaks with different sizes and locations as well as pre-transient pressure can produce the same pressure damping, and the corresponding pressure signals differ only in the first phase of the transient.

Within TTBTs, the manoeuvre that generates the transient is one of the most important features affecting the performance of the procedure. In other words, the more appropriate the manoeuvre (as specified immediately below), the more effective the TTBT. The first requirement of the manoeuvre is the shape of the pressure wave generated. More precisely, the sharper the pressure wave, the more accurate the identification of the fault, particularly in terms of its location. Consequently, slow manoeuvres are ineffective, and the valve must be closed/opened as quickly as possible. At the same time, to avoid the generation of large overpressures, the pre-transient mean velocity must be adequately small. As a consequence, small-diameter valves are used whether the pressure wave is generated by the Portable Pressure Wave Maker (PPWM) [42] or a side discharge valve [43]. Although manually operated valves can be an option, after checking the ease of the manoeuvre and therefore the speed, pneumatic-driven automatic valves are the most commonly used devices in this regard. Such devices are activated by a pneumatic rotary actuator connected to a closing port. Various geometries can be used for the closing port (valve body) interacting with the flow, the most common being the butterfly and ball configurations. In addition, the automatic valves better ensure the repeatability of the tests compared to manual valves. The second requirement of the manoeuvre is the stability of the pressure signal generated after the manoeuvre. “Spurious” pressure oscillations—e.g., those caused by an unstable valve body—contaminate the pressure signal and can obscure the pressure wave reflected by faults/singularities. The third requirement of the manoeuvre, less important than the first two, concerns the practicality of using the valve. In particular, if the characteristics of the manoeuvre do not depend significantly on the pre-transient flow conditions, and, in the case of pneumatically operated valves, on the value of the air pressure $p_{air}$, the performance of the tests will be greatly facilitated, especially in real pipe systems.

These premises form the basis of the present work. In fact, the choice of the right valve and test conditions is a fundamental task in achieving the optimal application of TTBTs [44,45]. The technology and application of automatic valves in water pipeline control are now established and growing with the new IoT approaches [46]. However, the relationship between the speed of actuation, the port geometry and the quality of the generated pressure waves deserves further investigation. The influence of the geometric characteristics of the valve body on the transient characteristics has been analysed in [47]. This paper describes the results of laboratory experiments carried out on steel and polyethylene pipelines subjected to water hammer events due to valve closure. Differences between transients generated by ball valves and butterfly valves are highlighted. The effects of different closing times and closure laws on the pressure waves generated by the water hammer are examined in [48]. The effect of six different types of valves (i.e., butterfly, cone, globe, diaphragm weir, full bore ball and plug) has been analysed in [49]. Some of these valves (i.e., diaphragm, plug and globe) produce less drastic changes in flow at the beginning and end of the closure; on the contrary, the others (i.e., butterfly, cone and full bore ball) produce more significant changes at the beginning of the closure.

The aim of this work is to investigate the effects of the geometry and operating conditions of automatic pneumatic-driven valves through laboratory tests using innovative measurement techniques. Particular attention is paid to the characteristics of the generated pressure wave (i.e., sharpness and stability) and the role of the air pressure, as well as the valve geometry. As discussed below, the types of valves and pipes considered in the experiments do not limit the significance of the results obtained. In fact, the proposed methodology can be applied to any other type of valve and any other type of pipeline, with the pipe pressurisation being the only indispensable requirement for the successful use of TTBTs. Accordingly, this paper is structured as follows. Section 2 describes the experimental setup and test conditions. Section 3 illustrates the procedure used to acquire the camera data used to monitor the valve displacement. Section 4 presents the analysis of the data in terms of valve displacement and pressure in order to compare the behaviour of the valves as they are affected by the test conditions. Finally, conclusions are drawn in Section 5.

## 2. Experimental Setup

**Figure 1:**
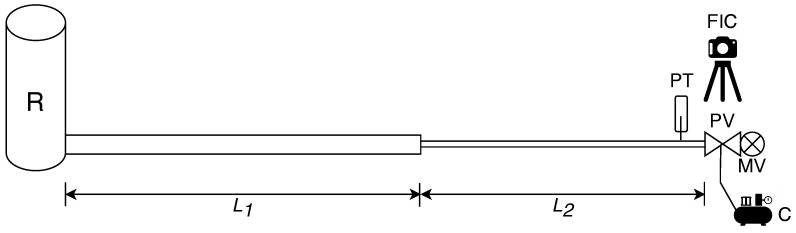
Sketch of the experimental setup (R = reservoir, PV = automatic pneumatic-driven valve (manoeuvre valve), MV = manual valve (regulation valve), C = air compressor, PT = pressure transducer and FIC = Fast Industrial Camera).

The experimental setup is arranged at the Water Engineering Laboratory (WEL) of the University of Perugia, Italy. It consists of an upstream reservoir, R, and a DN110 high-density polyethylene (HDPE) pipe supplying a DN22 copper pipe (Figure 1). The HDPE pipe has an internal diameter $D_{1}$ = 93.3 mm and length $L_{1}$ = 41.3 m, while the copper pipe has $D_{2}$ = 20 mm and $L_{2}$ = 30 m. At the downstream end section of the copper pipe, an automatic pneumatic-driven valve (PV) is installed to generate transients. The PV is in series with a manual ball valve (MV), which is used to regulate the pre-transient discharge $Q_{0}$, with the subscript 0 referring quantities to the pre-transient conditions. It is worth noting that the quite small length of the laboratory pipe makes transient tests more difficult with respect to real TMs, which are characterised by a much larger length (i.e., in the order of kilometres). In fact, the longer the pipe, the longer the duration of the valve manoeuvre $T_{c}$ can be to exclude the influence of the upstream boundary [50].

A pressure measurement section is arranged with a piezoresistive pressure transducer (DRUCK Series5000), indicated as PT at 0.15 m upstream of the PV. The discharge is measured by means of an ultrasonic flow meter installed in the copper pipe.

Two types of PV are used, i.e., a butterfly valve and a ball valve. It is worth noting that none of the phases of the proposed procedure is influenced by the valve characteristics. In the executed tests, the effect of air pressure $p_{air}$, provided to PV by a compressor (C), is investigated by considering two different values (i.e., 4 and 8 bar).

For each PV and $p_{air}$, three tests are carried out by setting $Q_{0}$ equal to 0.11, 0.09 and 0.03 L/s, respectively. This allows exploration of different pre-transient flow regimes in the copper pipe according to the value of the pre-transient Reynolds number ($Re_{2,0}$ = $\frac{Q_{0}D_{2}}{\nu A_{2}}$, where $A_{2}$ = copper pipe cross-sectional area and ν = kinematic viscosity). For the mentioned $Q_{0}$ values, the corresponding values of $Re_{2,0}$ are 7000, 5700 and 1900, respectively. Such values indicate that both laminar and turbulent flow are explored.

The PV motion is monitored by means of a Fast Industrial Camera (FIC), produced by IMAGINGSOURCE (Germany), model DMK 37BUX287 (720 × 540, up to 539 fps).

## 3. The Method for Measuring the Valve Displacement

**Figure 2:**
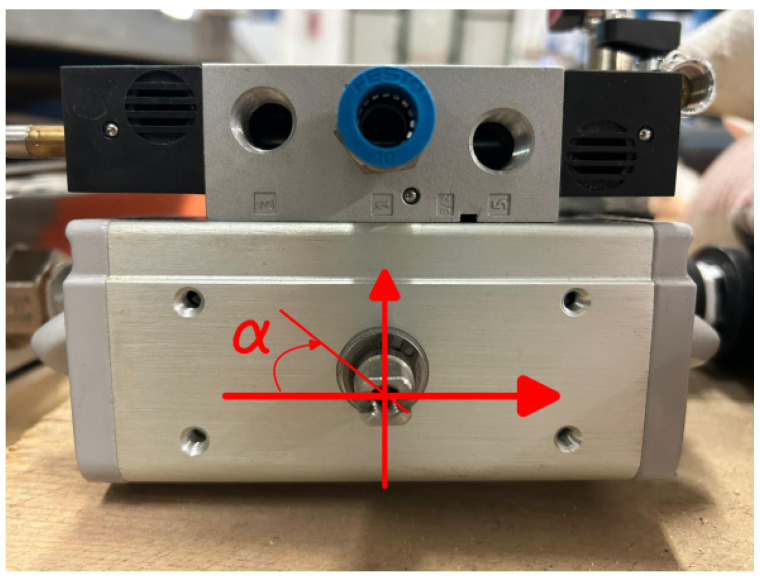
Automatic pneumatic-driven butterfly valve (by FESTO Group, Esslingen, Germany) with the reference system in the open configuration.

The primary objective of this study is to use a non-intrusive method for estimating valve opening based on image correlation, following the approach of Natili et al. [51], for wind turbine tachometer applications. This approach relies on the ability to track the valve position from images of the shaft protruding from the valve body as the groove on the shaft indicates the valve position. Figure 2 shows a top view of the valve with the aforementioned groove and reference system for measuring the angle. During the closing manoeuvre, the pin rotates 90° clockwise (the valve in the figure is in the open position).

The schematic procedure of the algorithm used to measure valve travel includes the following steps:

1. Capture a reference frame where the shaft pin is clearly visible;
2. Record a video sequence while the valve is moving;
3. Split the recorded video into a series of frames and convert them to greyscale;
4. Apply frame correction to remove pipeline and valve body vibrations using an iterative algorithm based on image correlation;
5. Implement the actual correlation algorithm by comparing all the frames to the reference, resulting in an output representing the degree of valve closure;
6. Assign a numerical value to each point of the signal and finally normalise the degree of opening from 1 (fully open) to 0 (fully closed).

In particular, the signal from the correlation algorithm ideally ranges from 1 (maximum correlation, image identical to the reference) to 0 (completely different from the reference image). The algorithm mentioned in step 5 is further described mathematically, as in [51].

**Figure 3:**
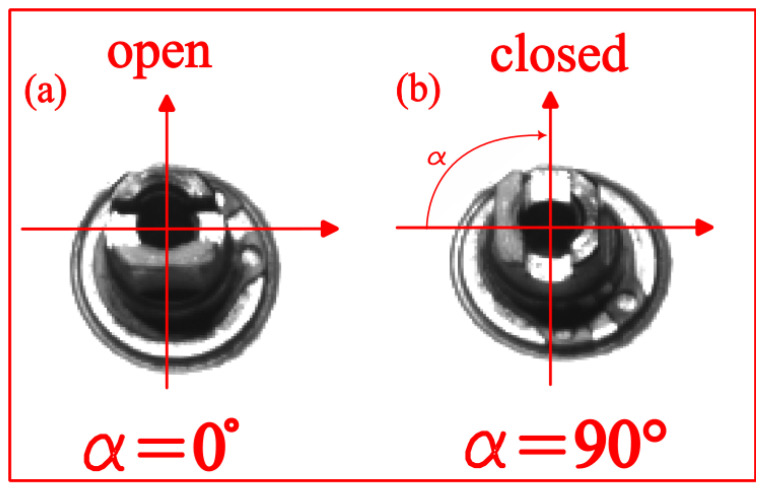
Schematic representation of the frames corresponding to the conditions before and at the end of the manoeuvre: (**a**) open position and (**b**) closed position.

Figure 3 shows the operations mentioned. In Figure 3a,b, the reference frame before and at the end of the manoeuvre is indicated, respectively, with α being the angle of the shaft connected to the closing port. Both are in black and white, and a cropping operation has been performed to ensure correct positioning on the pin.

**Figure 4:**
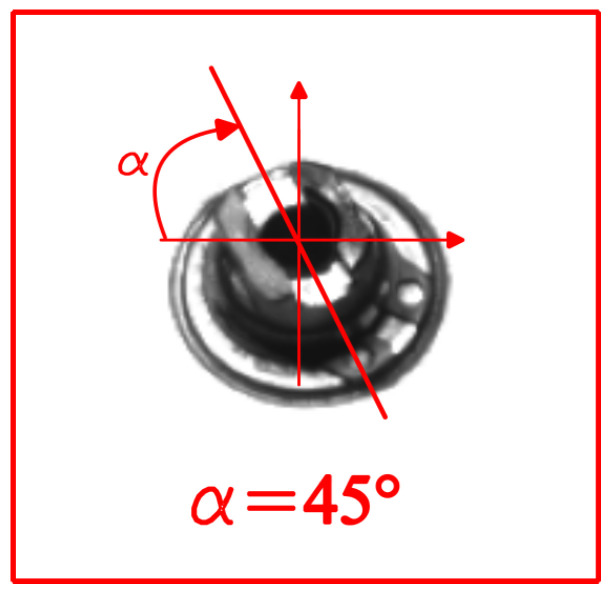
Schematic representation of the frames during the manoeuvre.

In Figure 4, a frame is shown corresponding to a point during the manoeuvre where the pin makes an angle of approximately 45° with the horizontal (0.5 according to the opening degree normalisation).

In a mathematical context, images are compared by evaluating the Pearson product–moment correlation coefficient. The images are initially acquired as 8-bit greyscale images using an *m* × *n* matrix which is then transformed into vectors of *m* × *n* dimensions.

Let $\hat{X}$ and $\hat{Y}$ be the matrices for the reference image and a sample frame from the video, both with dimensions of *m* × *n*, corresponding to the height and width of the chosen image resolution. Since the frames are captured in greyscale with a depth of 8 bits, the elements of the matrices essentially take values from 0 (representing black frames) to 255 (representing white frames).

Below are the notations for the representation of pixel matrices with $\hat{X}$ and $\hat{Y}$—Equation (Section 3)—and with *X* and Y as they appear after the transform operation as vectors of size (*m* × *n*) × 1—Equations (2) and (3).

(1) $$\hat{X}=\left[\begin{array}{cccc} x_{1,1} & \ldots & \ldots & x_{1,n}\\ \; & . & . & \\ \; & . & . & \\ \; & . & . & \\ x_{m,1} & \ldots & \ldots & x_{m,n} \end{array}\right]\;\;\;\;\;\;\;\;\;\;\;\;\;\hat{Y}=\left[\begin{array}{cccc} y_{1,1} & \ldots & \ldots & y_{1,n}\\ \; & . & . & \\ \; & . & . & \\ \; & . & . & \\ y_{m,1} & \ldots & \ldots & y_{m,n} \end{array}\right]$$

(2) $$X=\left[\begin{array}{cccc} x_{1}=x_{1,1} & x_{2}=x_{1,2} & \ldots & x_{m\;\times \;n}=x_{m,n} \end{array}\right]$$

(3) $$Y=\left[\begin{array}{cccc} y_{1}=y_{1,1} & y_{2}=y_{1,2} & \ldots & y_{m\;\times \;n}=y_{m,n} \end{array}\right]$$

Subsequently, by denoting $\bar{X}$ and $\bar{Y}$ as the mean values of *X* and Y, and given *m* and *n*, the elements of the covariance matrix C are calculated ($C_{1,1}$, $C_{2,2}$ and $C_{1,2}$ in Equations (4)–(6)), and then, from these values, the Pearson correlation coefficient ρ is determined by Equation (7) for each frame.

(4) $$C_{1,1}=var\left(X\right)=\frac{1}{nm-1}\sum_{i=1}^{nm}{\left(X_{i}-\bar{X}\right)}^{2}$$

(5) $$C_{2,2}=var\left(Y\right)=\frac{1}{nm-1}\sum_{i=1}^{nm}{\left(Y_{i}-\bar{Y}\right)}^{2}$$

(6) $$C_{1,2}=C_{2,1}=cov\left(X,Y\right)=\frac{1}{nm-1}\sum_{i=1}^{nm}\left(X_{i}-\bar{X}\right)\left(Y_{i}-\bar{Y}\right)$$

(7) $$\rho =\frac{C_{2,1}}{\sqrt{C_{1,1}C_{2,2}}}$$

Having obtained a video of the closing manoeuvre, converted it into a series of frames (knowing the acquisition time and camera frame rate) and applied the algorithm to all $Y_{i}$ frames, the output consists of a time series of correlation points at defined time intervals. As a final step (Figure 3), the boundary conditions, according to the normalisation, are defined as follows:

- $\alpha =0^{\circ }$ for ρ=1;
- $\alpha =90^{\circ }$ for ρ=0.

**Figure 5:**
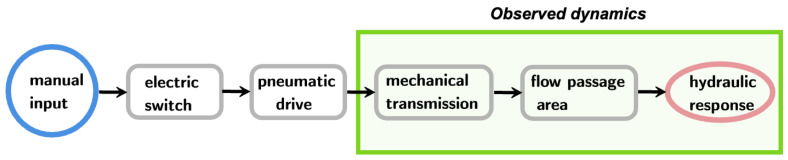
Schematic representation of the overall closing process.

Finally, it is possible to move from a time history of the Pearson coefficient (or its complement) to a time history of the angular displacement of the valve or, finally, to a normalised degree of opening. In this way, it is possible to observe how the closing of the valve evolves over time from a “mechanical” point of view. The whole closing process starts from a manual input on the electric switch (Figure 5), then goes through the pneumatic drive, the mechanical transmission and finally the flow area, which induces the hydraulic response.

The experimental equipment used in present work includes:

- The camera to record the mechanical movement of the valve;
- The pressure measurements for monitoring the hydraulic response.

In this way, in the present work, only the last part of the total opening process is analysed (the one enclosed by the green box in Figure 5).

## 4. Data Analysis

### 4.1. Valve Displacement Data

The results of the video post-processing were used to analyse the characteristics of the PV closing time history under different operating conditions.

The first step was to determine the accuracy of the valve position measurements. Several tests were carried out with exactly the same setup, and then the closing time histories were compared. The first time step considered was the one that gave a normalised closing of 1% (opening degree < 0.99) so that two different events could be synchronised. A direct comparison of the repeated transient closures gave a good coefficient of determination (close to 1), corresponding to a maximum error of 3.6% for the butterfly valve and 4% for the ball valve. These figures confirm the robustness of the approach and the reliability of the measurements.

The effects of pre-transient flow, $Q_{0}$, and air pressure, $p_{air}$, on the performance of the two PVs were then investigated.

**Figure 6:**
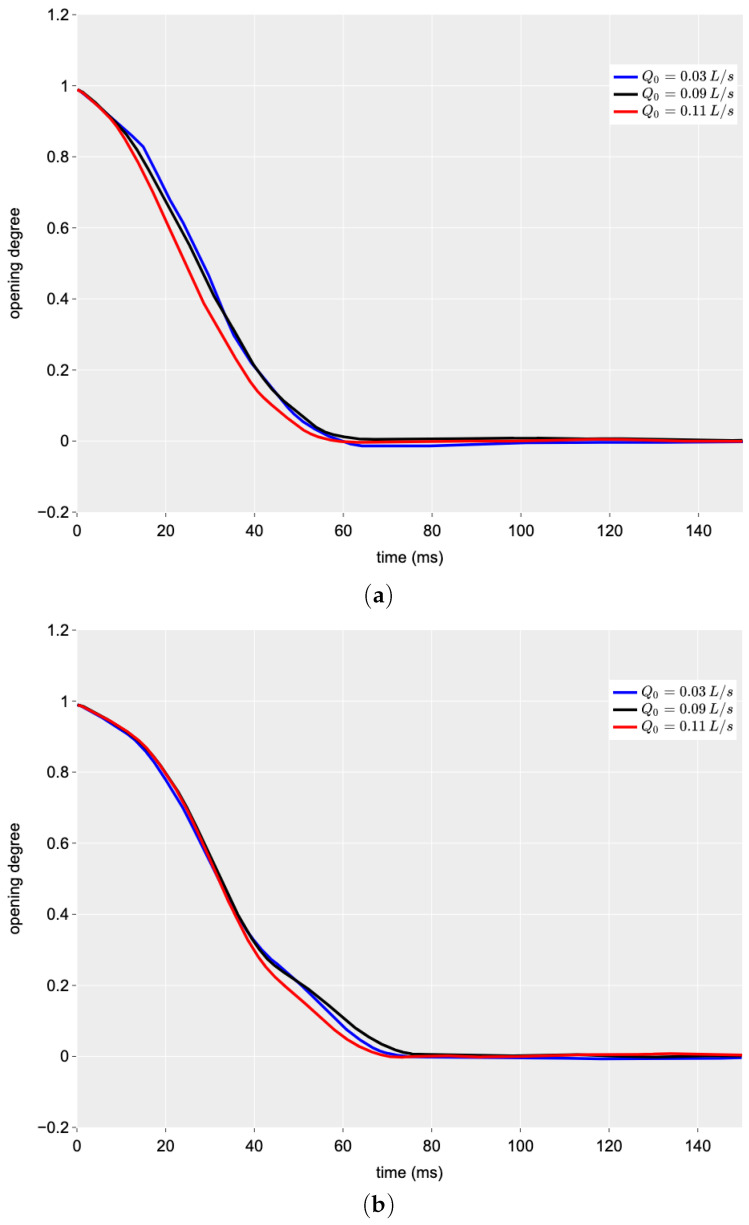
Effect of the discharge, $Q_{0}$, on the closing manoeuvre time history ($p_{air}$ = 8 bar): (**a**) butterfly valve and (**b**) ball valve.

In Figure 6, the influence of $Q_{0}$ on the operation of the butterfly and ball PVs is analysed for a given value of $p_{air}$. Both configurations showed a low sensitivity to flow, with a slightly faster closure observed at higher flows. The butterfly valve (see Figure 6a) appears to have a higher sensitivity to flow, with faster closures at high flow rates. This was expected because the butterfly has a higher surface area interacting with the flow, and, as a result, the closure is somehow “assisted” by the flow.

**Figure 7:**
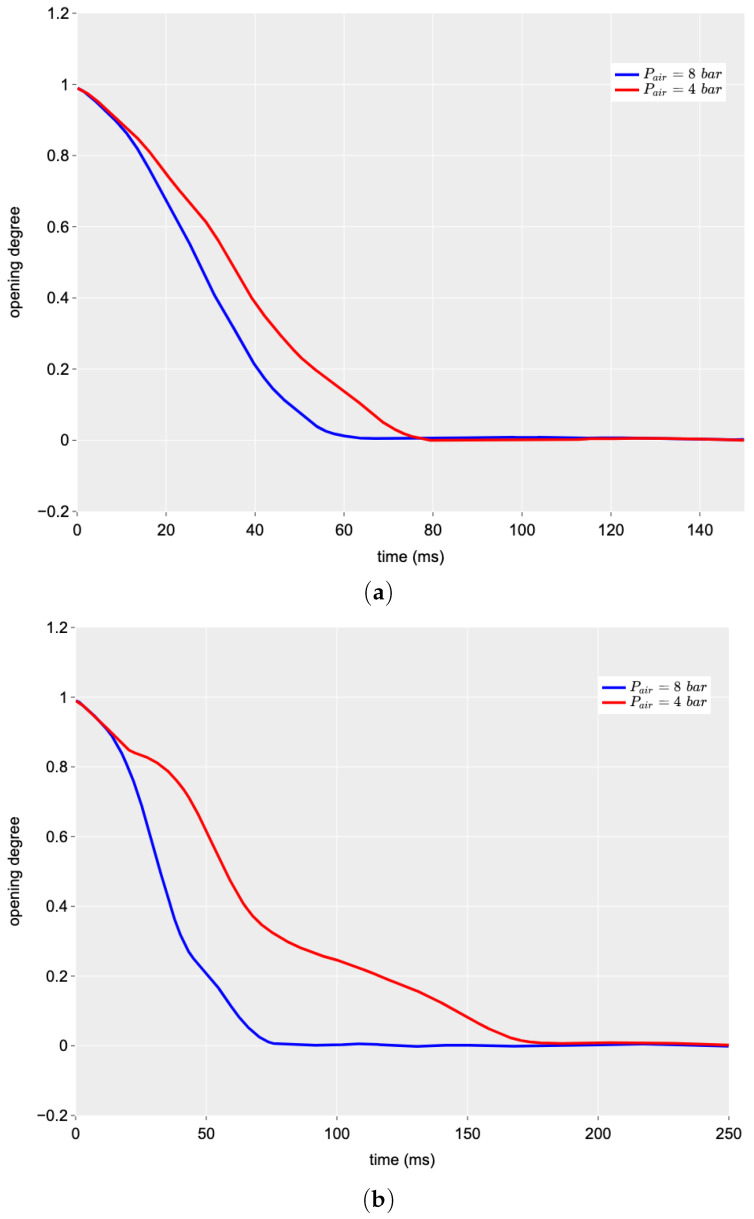
Effect of the air pressure, $p_{air}$, on the closing manoeuvre time history ($Q_{0}$ = 0.09 L/s): (**a**) butterfly valve and (**b**) ball valve.

Figure 7 analyses the effect of air pressure in the pneumatically driven system. Two different levels of air pressure were used in the tests: 8 bar (the standard recommended pressure for the valve) and 4 bar (a lower level that can drive the system anyway). In this case, the behaviour of the two valve geometries was very different. In both configurations, the total time taken to close completely was longer when using air at the lower pressure ($p_{air}$ = 4 bar), but this tendency was much more pronounced in the case of the ball valve.

For the butterfly valve, the time for a complete closing (i.e., the duration of the manoeuvre), $T_{c}$, changed from 60 ms at $p_{air}$ = 8 bar to 75 ms at $p_{air}$ = 4 bar with an increment of 24%. On the contrary, for the ball valve, $T_{c}$ was equal to 74 ms at $p_{air}$ = 8 bar and 175 ms at $p_{air}$ = 4 bar with an increment of 136%. This result seems to be due to the different geometry of the closing port; in the case of the ball valve, the friction between the two globes used for adjusting the discharge area is very important, especially when the drive action, due to the pneumatic system, is weak as an effect of the lower air pressure. It is worth noting that all $T_{c}$ values allow exclusion of any interference of the upstream boundary condition on the characteristics of the generated pressure wave. This noticeably facilitates the analysis of the pressure signal within TTBTs. In real TMs that are characterised by a much larger length, such values of $T_{c}$ are even more adequate.

**Figure 8:**
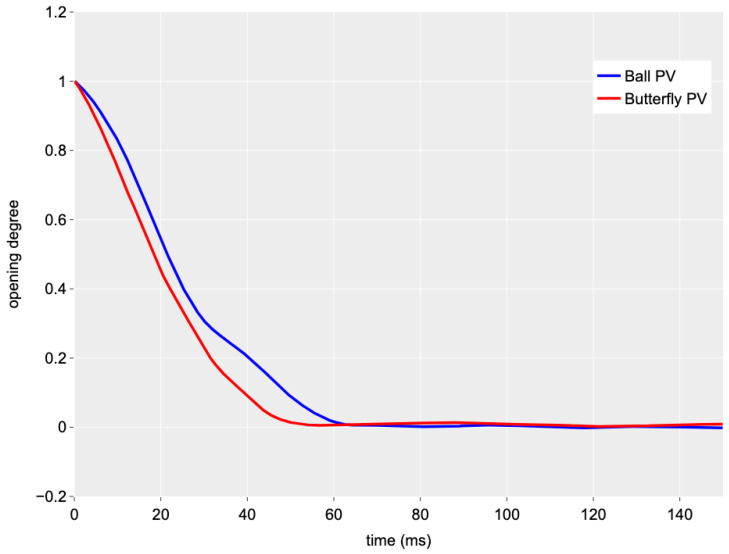
Comparison of the time history of the valve opening degree for the two PVs ($Q_{0}$ = 0.09 L/s and $p_{air}$ = 8 bar).

A direct comparison of the operation of the two valve geometries can be performed with regard to the air pressure $p_{air}$ = 8 bar. In Figure 8, the time history of the valve opening degree for the closing manoeuvre for the two valves is represented. It is quite clear that the ball valve needs more time for a complete closing compared to the butterfly one; the two values of $T_{c}$ are 74 ms (ball) and 60 ms (butterfly), respectively. This implies a value of $T_{c}$ that is 23% higher for the ball valve.

A mathematical model has been developed to characterise the dynamic behaviour of the system using displacement data processing. It has been found that a third-order model, in accordance with the theory of a pneumatic actuator, reproduces the behaviour of the system in a reliable way. The transfer function of the studied model has the following form:

(8) $$H\left(s\right)=\frac{1}{as^{3}+bs^{2}+cd+d}$$

where a,b,c and *d* are the identified parameters, and *s* is the complex Laplace’s variable. Different model settings were analysed, and it was found that the third-order model is the optimal configuration for obtaining a high value of R-squared. This confirms the robustness of the proposed measurement approach and represents a first step towards a deeper investigation of the dynamics of the system through identification.

### 4.2. Pressure Data

For the short period analysis of pressure signals, it is crucial to focus on the pipe first characteristic time, τ, i.e., the time interval taken by the generated pressure wave to travel along the pipe and be reflected back to the transient generation point. Accordingly, in this paper, as attention was focused on the copper pipe, the considered time interval was equal to about $\tau_{2}=2L_{2}/a_{2}$, where $a_{2}$ (≃1220 m/s) is the pressure wave speed of the copper pipe. Since the copper pipe was not affected by any fault or singularity, in the first characteristic time, the pressure signal at the measurement section was expected to be quite “stable”, i.e., with no significant pressure variation, until the arrival of the negative pressure wave reflected by the series junction between the copper and HDPE pipes. Nevertheless, the experimental pressure signals were affected by some pressure variations that can be ascribed in part to noise and vibrations and in part to the PV behaviour.

**Figure 9:**
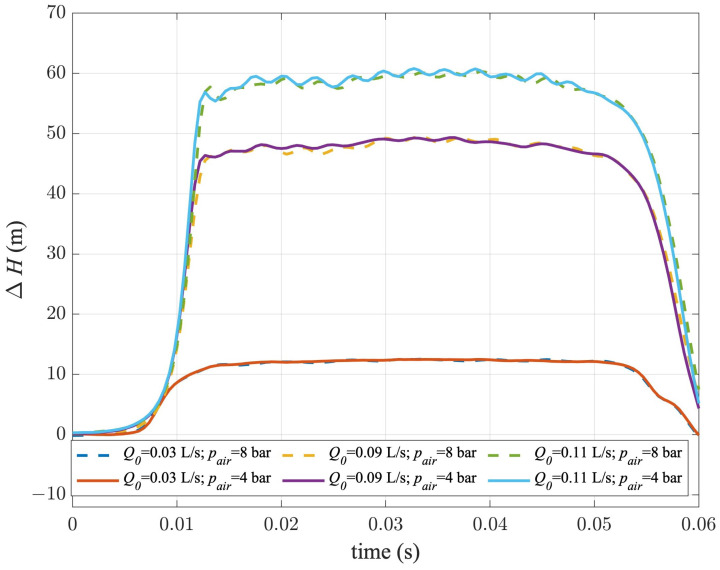
Pressure signals acquired during transient tests carried out with the butterfly PV for the three chosen values of $Q_{0}$ and two values of $p_{air}$.

Pressure signals acquired during transient tests carried out with the butterfly PV are shown in Figure 9, where the pressure variation $\Delta H=H-H_{0}$ is plotted for the first tens of milliseconds of the transient event, with *H* being the piezometric head. As expected, the three values of $Q_{0}$ give rise to different values of the generated overpressure, according to the Allievi–Joukovski formula [50,52]. However, the larger the $Q_{0}$, the larger the friction effect, and then the larger the maximum value of the pressure. It is worth noting that, for each couple of tests carried out for a given value of $Q_{0}$, the pressure wave seemed not be influenced by the air pressure. In fact, the pressure increased due to the PV manoeuvre, almost overlapping in terms of wave sharpness. After such a pressure increase, *H* remained quite constant, as expected in a singularity-free pipe. However, this portion of the pressure signal exhibited a different “stability” degree depending on the $Q_{0}$ value. In fact, it can be noted that, for the pressure signals acquired for $Q_{0}$ = 0.11 L/s, the pressure oscillations were more significant than in the other tests.

**Figure 10:**
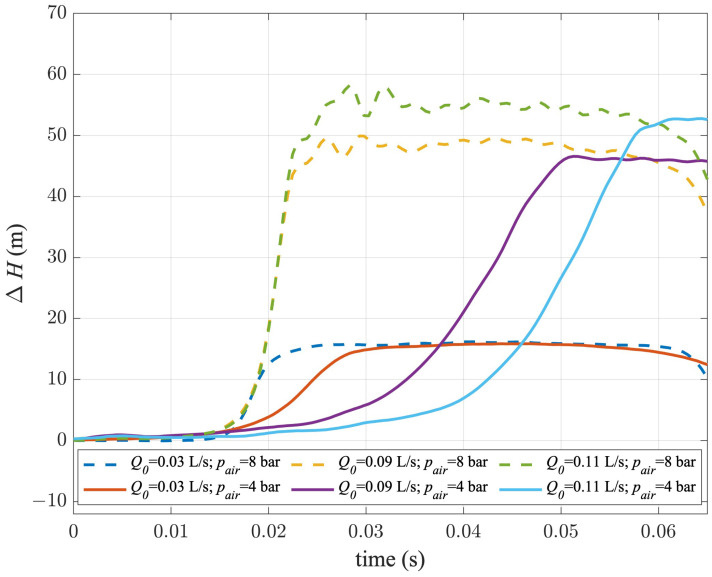
Pressure signals acquired during transient tests carried out with the ball PV for the three chosen values of $Q_{0}$ and two values of $p_{air}$.

Pressure signals acquired during transient tests carried out with the ball PV are shown in Figure 10. At first sight, some differences with respect to the butterfly PV can be observed. In fact, pressure signals acquired for tests at $p_{air}$ = 8 bar were characterised, as for the butterfly PV, by a very similar sharpness of the pressure wave. On the contrary, for $p_{air}$ = 4 bar, the wave front was much less sharp, and this effect was larger the higher the value of $Q_{0}$.

**Figure 11:**
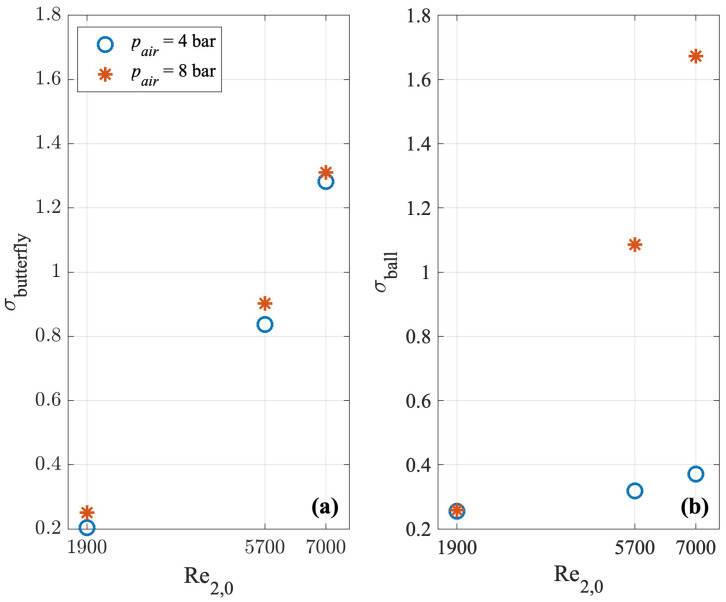
Standard deviation, σ, of the pressure signal during the copper pipe’s first characteristic time for manoeuvres made by (**a**) the butterfly PV and (**b**) the ball PV for the three chosen values of $Q_{0}$ and two values of $p_{air}$.

To better understand if and how the valve geometry and the manoeuvre parameters affect the pressure signals, the standard deviation σ was evaluated considering the part of the pressure signal after the onset of the generated overpressure. The values of $\sigma_{butterfly}$ and $\sigma_{ball}$ are shown in Figure 11a,b, respectively, for the three values of the pre-transient Reynolds number and air pressure.

It can be noted that the increase in the pre-transient discharge implies a larger value of the standard deviation, for both the PVs. Moreover, the role played by the air pressure seems to have had a larger relevance in the case of the ball PV compared to the butterfly one.

## 5. Conclusions

The use of fast transients for fault detection in long transmission mains (TMs)—the so-called Transient Test-Based Techniques (TTBTs)—makes the generation of controlled transients crucial. In order to maximise the information that can be extracted from the measured pressure signal, the transients must meet certain requirements. In particular, the manoeuvre that generates the transient must be fast and repeatable. This implies the use of small-diameter valves and often pneumatic-driven operated automatic valves. In addition, the pressure wave generated must be as sharp and stable as possible.

In this paper, experimental transient tests were carried out at the Water Engineering Laboratory (WEL) of the University of Perugia. Transients were generated in a copper pipe by closing two types of automatic pneumatic-driven valves (i.e., a butterfly valve and a ball valve). The effects of the pre-transient discharge and the valve air pressure were studied in terms of the sharpness of the pressure wave generated and the stability of the pressure signal during the first phase of the transient.

While the pressure in the immediate vicinity of the pneumatic valve was measured using a piezoresistive pressure transducer, a Fast Industrial Camera was used to monitor the valve displacement. Video processing was used to extrapolate the time history of the opening degree for each configuration analysed. The results show that the closing time as well as the trend of the opening degree time history can be affected by the operating conditions (in terms of discharge and air pressure applied to the PV); however, different behaviours were also noted for the two valve geometries. In particular, the air pressure had a much greater effect on the ball PV than on the butterfly PV, with an increase of 185% in the closing time when the pressure was changed from the nominal level of 8 bar to 4 bar. This result is somehow in line with the different architecture of the shut-off orifice; in the case of the ball valve, the two sliding balls that adjust the outlet area cause significant friction so that, at the lower pressure level, the dynamics of the system are compromised. This is not the case for the butterfly valve, which is characterised by a lower friction area. The differences in valve closing are then reflected in the pressure waves through the variation in closing time and effective discharge area (according to the different geometries of the closing orifices).

The results of the tests show that, for the butterfly valve, the sharpness of the pressure waves is not significantly influenced by the air pressure. Moreover, an increase in the pre-transient discharge implies the presence of more spurious pressure oscillations in terms of the stability of the pressure signal. For the ball valve, the air pressure has a significant effect on the sharpness of the pressure wave; the lower the air pressure, the slower the pressure rise. For the butterfly valve, the increase in the pre-transient discharge reduces the stability of the pressure signal. For the highest pressure value, which implies a better performance in terms of pressure wave sharpness, the use of the ball valve implies a much lower stability of the pressure signal.

The present work has allowed the investigation of the influence of the valve geometry as well as the operating conditions on the characteristics of the generated pressure waves within TTBTs. Future work will focus more on the influence of the time delays for the closing manoeuvre with automatic PV valves. This ambitious task can only be developed by improving the quality and number of parameters monitored during the experimental campaign. A major improvement will also be made possible by synchronising the video recordings with the pressure measurements. This will be essential for capturing the correct timing and developing a deeper analysis of the influence of the different valve geometries.

## Data Availability

Experimental data are available upon request.

## References

[1] Hunaidi O., Chu W.T. (1999). Acoustical characteristics of leak signals in plastic water distribution pipes. Appl. Acoust..

[2] Almeida F., Brennan M., Joseph P., Whitfield S., Dray S., Paschoalini A. (2014). On the acoustic filtering of the pipe and sensor in a buried plastic water pipe and its effect on leak detection: An experimental investigation. Sensors.

[3] Abdulshaheed A., Mustapha F., Ghavamian A. (2017). A pressure-based method for monitoring leaks in a pipe distribution system: A Review. Renew. Sustain. Energy Rev..

[4] Laven K., Lambert A. What do we know about real losses on transmission mains. Proceedings of the IWA Specialised Conference “Water Loss”.

[5] Datta S., Sarkar S. (2016). A review on different pipeline fault detection methods. J. Loss Prev. Process. Ind..

[6] Ma Q., Tian G., Zeng Y., Li R., Song H., Wang Z., Gao B., Zeng K. (2021). Pipeline in-line inspection method, instrumentation and data management. Sensors.

[7] Dobmann G., Barbian O., Willems H. (2007). State of the art of in-line nondestructive weld inspection of pipelines by ultrasonics. Russ. J. Nondestruct. Test..

[8] Parlak B.O., Yavasoglu H.A. (2023). A comprehensive analysis of in-line inspection tools and technologies for steel oil and gas pipelines. Sustainability.

[9] Chen H., Zhang X., Li J. Ultra low frequency electromagnetic wave localization and application to pipeline robot. Proceedings of the 2006 IEEE/RSJ International Conference on Intelligent Robots and Systems.

[10] Kazeminasab S., Sadeghi N., Janfaza V., Razavi M., Ziyadidegan S., Banks M.K. (2021). Localization, mapping, navigation, and inspection methods in in-pipe robots: A review. IEEE Access.

[11] Brockhaus S., Ginten M., Klein S., Teckert M., Stawicki O., Oevermann D., Meyer S., Storey D. (2014). In-line inspection (ILI) methods for detecting corrosion in underground pipelines. Underground Pipeline Corrosion.

[12] Mohapatra P.K., Chaudhry M.H., Kassem A., Moloo J. (2006). Detection of partial blockages in a branched piping system by the frequency response method. J. Fluids Eng..

[13] Colombo A., Lee P., Karney B.W. (2009). A selective literature review of transient-based leak detection methods. J. Hydro-Environ. Res..

[14] Liu Z., Kleiner Y. (2013). State of the art review of inspection technologies for condition assessment of water pipes. Measurement.

[15] Xu X., Karney B. (2017). An overview of transient fault detection techniques. Modeling and Monitoring of Pipelines and Networks.

[16] Ayati A.H., Haghighi A., Lee P.J. (2019). Statistical review of major standpoints in hydraulic transient-based leak detection. J. Hydraul. Struct..

[17] Duan H., Pan B., Wang M., Chen L., Zheng F., Zhang Y. (2020). State-of the-art review on the transient flow modeling and utilization for urban water supply system (UWSS) management. J. Water Supply Res. Technol.-Aqua.

[18] Che T.C., Duan H.F., Lee P.J. (2021). Transient wave-based methods for anomaly detection in fluid pipes: A review. Mech. Syst. Signal Process..

[19] Brunone B., Maietta F., Capponi C., Keramat A., Meniconi S. (2022). A review of physical experiments for leak detection in water pipes through transient tests for addressing future research. J. Hydraul. Res..

[20] Brunone B., Maietta F., Capponi C., Duan H.F., Meniconi S. (2023). Detection of partial blockages in pressurized pipes by transient tests. A review of the physical experiments. Fluids.

[21] Misiunas D., Vítkovský J., Olsson G., Simpson A., Lambert M. (2005). Pipeline break detection using pressure transient monitoring. J. Water Resour. Plan. Manag..

[22] Capponi C., Martins N.M., Covas D.I.C., Brunone B., Meniconi S. (2024). Transient test-based techniques for checking the sealing of in-line shut-off valves and capturing the effect of series junctions—Field tests in a real pipe system. Water.

[23] Liou C. (1998). Pipeline leak detection by impulse response extraction. J. Fluids Eng..

[24] Covas D., Ramos H. (2010). Case studies of leak detection and location in water pipe systems by inverse transient analysis. J. Water Resour. Plan. Manag..

[25] Taghvaei M., Beck S., Boxall J. (2010). Leak detection in pipes using induced water hammer pulses. Int. J. COMADEM.

[26] Shucksmith J.D., Boxall J.B., Staszewski W.J., Seth A., Beck S.B.M. (2012). Onsite leak location in a pipe network by cepstrum analysis of pressure transients. J. Am. Water Work. Assoc..

[27] Ghazali M., Staszewski W., Shucksmith J., Boxall J., Beck S. (2011). Instantaneous phase and frequency for the detection of leaks and features in a pipeline system. Struct. Health Monit..

[28] Sun J., Wang R., Duan H.F. (2016). Multiple-fault detection in water pipelines using transient-based time-frequency analysis. J. Hydroinformatics.

[29] Wang X.J., Lambert M.F., Simpson A.R. (2005). Detection and location of a partial blockage in a pipeline using damping of fluid transients. J. Water Resour. Plan. Manag..

[30] Liggett J.A., Chen L.C. (1994). Inverse transient analysis in pipe networks. J. Hydraul. Eng..

[31] Tuck J., Lee P. (2013). Inverse transient analysis for classification of wall thickness variations in pipelines. Sensors.

[32] Soares A.K., Covas D.I., Reis L.F.R. (2011). Leak detection by inverse transient analysis in an experimental PVC pipe system. J. Hydroinformatics.

[33] Keramat A., Wang X., Louati M., Meniconi S., Brunone B., Ghidaoui M.S. (2019). Objective functions for transient based pipeline leakage detection in a noisy environment: Least square and matched-filter. J. Water Resour. Plan. Manag..

[34] Mpesha W., Gassman S.L., Chaudhry M.H. (2001). Leak detection in pipes by frequency response method. J. Hydraul. Eng..

[35] Lee P.J., Vítkovskỳ J.P., Lambert M.F., Simpson A.R., Liggett J.A. (2005). Frequency domain analysis for detecting pipeline leaks. J. Hydraul. Eng..

[36] Sattar A.M., Chaudhry M.H., Kassem A.A. (2008). Partial blockage detection in pipelines by frequency response method. J. Hydraul. Eng..

[37] Duan H.F., Lee P.J., Kashima A., Lu J., Ghidaoui M.S., Tung Y.K. (2013). Extended blockage detection in pipes using the system frequency response: Analytical analysis and experimental verification. J. Hydraul. Eng..

[38] Gong J., Simpson A., Lambert M.F., Zecchin A.C., Kim Y.I., Tijsseling A.A. (2013). Detection of distributed deterioration in single pipes using transient reflections. J. Pipeline Syst. Eng. Pract..

[39] Gong J., Lambert M.F., Nguyen S.T.N., Zecchin A., Simpson A.R. (2018). Detecting thinner-walled pipe sections using a spark transient pressure wave generator. J. Hydraul. Eng..

[40] Brunone B., Meniconi S., Capponi C. (2023). The damping of pressure peaks during transients for fault detection in pressurized pipelines. An expeditious and manager-oriented diagnosis procedure. J. Hydraul. Eng..

[41] Capponi C., Meniconi S., Lee P.J., Brunone B., Cifrodelli M. (2020). Time-domain analysis of laboratory experiments on the transient pressure damping in a leaky polymeric pipe. Water Resour. Manag..

[42] Brunone B., Capponi C., Meniconi S. (2021). Design criteria and performance analysis of a smart portable device for leak detection in water transmission mains. Measurement.

[43] Meniconi S., Brunone B., Tirello L., Rubin A., Cifrodelli M., Capponi C. (2024). Transient tests for checking the Trieste subsea pipeline: Toward field tests. J. Mar. Sci. Eng..

[44] Wood D.J., Jones S.E. (1973). Water-hammer charts for various types of valves. J. Hydraul. Div..

[45] Azoury P.H., Baasiri M., Najm H. (1986). Effect of valve-closure schedule on water hammer. J. Hydraul. Eng..

[46] Safta C.A., Cococi V.N., Călinoiu C., Marin A. (2023). Performance of water valves required by water supply network digitalization process. IOP Conf. Ser. Earth Environ. Sci..

[47] Kodura A. (2016). An analysis of the impact of valve closure time on the course of water hammer. Arch. Hydro-Eng. Environ. Mech..

[48] Han Y., Shi W., Xu H., Wang J., Zhou L. (2022). Effects of closing times and laws on water hammer in a ball valve pipeline. Water.

[49] Al-Zaidi B.M., Ismaeel A.J. (2022). Effect of hydraulic characteristics on fluid transients analysis under different types of control valves. J. Ecol. Eng..

[50] Swaffield J., Boldy A. (1993). Pressure Surges in Pipe and Duct Systems.

[51] Natili F., Castellani F., Astolfi D., Becchetti M. (2020). Video-tachometer methodology for wind turbine rotor speed measurement. Sensors.

[52] Wylie E.B., Streeter V.L. (1993). Fluid Transients in Systems.

